# Short Communication: Use of Infrared Thermometers for Cutaneous Temperature Recording: Agreement with the Rectal Temperature in *Felis catus*

**DOI:** 10.3390/ani12101275

**Published:** 2022-05-16

**Authors:** Claudia Giannetto, Giuseppe Acri, Melissa Pennisi, Giuseppe Piccione, Francesca Arfuso, Annastella Falcone, Elisabetta Giudice, Simona Di Pietro

**Affiliations:** 1 Department of Veterinary Sciences, Polo Universitario dell’Annunziata, University of Messina, 98168 Messina, Italy; melissa-10@hotmail.com (M.P.); gpiccione@unime.it (G.P.); farfuso@unime.it (F.A.); afalcone@unime.it (A.F.); egiudice@unime.it (E.G.); dipietros@unime.it (S.D.P.); 2 Department of Biomedical, Dental, Morphological and Functional Imaging Sciences, University of Messina, 98125 Messina, Italy; gacri@unime.it

**Keywords:** non-contact infrared thermometers, skin temperature, body temperature, cats

## Abstract

**Simple Summary:**

The recording of body temperature by rectal temperature assessments is a stressful procedure for cats. For this purpose, alternative methods for using rectal digital thermometers to monitor body temperature were investigated. Skin temperature was recorded in 20 cats, in 5 different body regions, and compared with the rectal temperature. The obtained data indicated that the cutaneous temperature recorded by the infrared thermometers was not in agreement with the data recorded by the digital thermometer in the rectum.

**Abstract:**

In veterinary medicine, the gold standard for assessing body temperature is rectal temperature assessment. Considering that this procedure is stressful for many species, in particular for cats, it could be clinically important to consider an alternative approach for the monitoring of core body temperature. The aim of this study was to test if cutaneous temperature measurements by means of different infrared thermometers are in agreement with the most commonly used method for body temperature measurement in cats. The cutaneous temperature was recorded in the jugular, shoulder, rib, flank, and inner thigh, using three different non-contact infrared thermometers (IR1, IR2, and IR3) in 20 cats. The cutaneous temperature was then compared to the rectal temperature, recorded by means of a digital thermometer. The obtained data indicated that the cutaneous temperature recorded by the infrared thermometers was not in agreement with the data recorded by the digital thermometer in the rectum. In cats, the use of non-contact infrared thermometers gave no reproducible or constant data to justify their application for the recording of body temperature instead of rectal temperature recording. In addition, the infrared temperature measurement devices generated results that were not in good agreement among themselves, providing a novel result of clinical importance.

## 1. Introduction

Temperature measurement is an important step in clinical examinations of animals because there is an evident association between temperature and disease [1], and it represents a valuable tool for monitoring the physiological status, welfare, and stress responses of animals [2]. The core body temperature is assessed by an invasive contact device, such as an esophageal or a pulmonary artery thermistor, although these are not suitable for conscious veterinary patients [3,4]. In veterinary medicine, rectal temperature measurement has traditionally been the most common method for assessing body temperature. However, obtaining the rectal temperature can be a stressful procedure for patients, particularly if serial measurements are needed [5,6]. Some authors showed that rectal temperature measurement could provoke defensive behaviors from the animal under examination [7]. This invasive procedure, in reactive and stressful animals, might alter the result of the measurement, thus compromising its interpretation and the consequent diagnostic and therapeutic measures [8]. In fact, anxiety and stress affect many clinical measurements used to evaluate animal health [9,10,11,12]. In particular, in homeothermic animals, it has been documented that acute and chronic stress are associated with a rise in the core body temperature [13]. In clinical environments, cats can experience considerable anxiety and stress because of unfamiliar surroundings, people, and methods of handling. Furthermore, the effects of stress on cats may persist far beyond the clinic visit. Evidence-based best-practice guidelines recommend low-stress handling procedures and reducing the use of invasive practices, replacing them with non-restrictive practices [14,15]. Modern measurement techniques are based on the use of diagnostic tools that reduce a subject’s stress by a quick assessment of the body temperature, without the need for restraint or contact [16]. 

In human medicine, body temperature measurement methods comprise conductive thermocouples, thermistors, and telemetry systems, as well as contact-free infrared thermometry and imaging [17,18]. Infrared (IR) methodologies determine the surface body temperature by measuring the natural electromagnetic radiation emitted from the body [2]. Several infrared thermometers have been developed specifically for veterinary use, resulting in better patient compliance and quicker results. The body temperature is highly variable and is influenced by a vast range of environmental, physiological, and pathological factors, including ambient humidity and temperature, and disease processes; indeed, the core body temperature brings about changes in the body temperature [2,5,6,7,8,18,19]. Thermal comfort is guaranteed by the transfer of heat by the cutaneous vasomotor adjustments that permit or reduce the convection between the body’s core and the skin surface through the bloodstream [20]. Skin temperature changes have also been associated with the vasoconstriction and vasodilatation of microcirculation due to an autonomic nervous system response to inflammation, infectious, neoplastic, pain-induced, or stress effects [19].

In human medicine, the measurement of the body temperature of a specific skin region—for example, the axillary temperature—seems to be the least stressful method for assessing temperature because of the minimal cooperation required, not only in children [21]. The surface body temperature can be determined using thermal windows, referring to regions with surface blood vessels where changes can be detected that represent surface circulation. Infrared thermography is used for the diagnosis of and follow-ups for several disorders, including vascular, orthopedic, neurologic, and neoplastic diseases that cause changes in the surface body temperature. Different skin regions were proposed in cats to assess superficial temperature [5,19], such as ocular, abdominal [22], dorsal and lateral views of the entire body and paw print to assess painful conditions [23], and pelvic limbs to detect aortic thromboembolism [24].

In adult and pediatric practice, an inconsistent correlation of infrared methodology with core or rectal temperatures was reported [25,26,27].

Alternative methods have been investigated in many mammalian species, in which the cutaneous temperature recorded in different body regions has been compared with rectal temperature. Non-contact infrared thermometry and thermography have been used in laboratory animals, such as rabbits [28], monkeys [29], guinea pigs [30], and mice [31], and in livestock [2,32,33,34], horses [33,35,36,37], and small animals [38,39,40], with conflicting data.

In dogs, the auricular temperature measured with an infrared thermometer was similar to the body temperature obtained with a rectal thermometer [41,42,43], also during the monitoring of hyperthermia induced by physical exercise [38]. The gum temperature exhibited the best clinical potential when digit, snout, axilla, eye, gum, inguinal region, and anal verge temperatures were compared to rectal temperature [44].

In cats, many studies have been conducted to validate the use of alternative methods for recording the body temperature and rectal temperature. The discussion of the clinical use of contact-free infrared thermometry has centered on various anatomical regions that could be used as sites for cutaneous temperature measurements, such as ear, pinna, preauricular area, tympanic membrane, nasal planum, gingival mucosa, eye, dorsal neck, lateral neck, ventral neck, thorax, axilla, metacarpal pad, abdomen, medial thigh, lateral thigh, tail, and perineum [5,6,7,45,46,47,48]. A marked surface temperature variation was shown in cats by a forward-looking infrared camera. Therefore, numerous studies compared the reliability and repeatability of skin temperature measurements at different sites [5].

Ocular temperature has been considered an alternative method for body temperature measurement. When ocular temperature is used, it is necessary to consider that the surface temperature of the eye is 1.2 °C less than the rectal temperature in cats [40]. In other body regions, the obtained results are discordant: some authors indicated the use of the auricular thermometer as a reliable alternative to rectal thermometry for the assessment of body temperature [43], while others reported that the axillary and tympanic membrane temperatures were not interchangeable with the rectal temperature [46]. 

Despite the large existing literature on infrared thermometry, a reference range for diverse body regions’ temperature measurements in cats needs to be validated. Moreover, there is an evident gap in the existing literature regarding the comparison of different IR thermometer models for measuring surface body temperature in cats, in order to verify their interchangeability. 

The aim of this study was to evaluate the use of non-contact infrared thermometers in healthy cats by the investigation of five body regions and by the comparison of three different non-contact infrared thermometers. The objective of the study was to verify if the cutaneous temperature measurement by means of different infrared thermometers is in agreement with rectal temperature, identifying which body regions and infrared devices could be more appropriate for this scope.

## 2. Materials and Methods

### 2.1. Animal and Experimental Design

Twenty client-owned cats of different breeds (Maine Coon, European Shorthair, and Ragdoll), 10 females and 10 neutered males, 3–5 years old, with a mean body weight 4.5 ± 0.20 kg, with a coat color from white to grey, and with coat lengths between short- and medium-hair were recruited for the study. Cats examined between March and June 2020 were eligible for inclusion. Cats suffering from ectoparasites, recto-anal disease, dermatitis, cutaneous neoplasia, and metabolic and contagious diseases were excluded, as were any cats that had a rectal temperature outside the range of 37.8 °C to 39.4 °C, classified as normothermia [41,46]. This work involved the use of non-experimental animals only (owned animals). The established, internationally recognized high standards (”best practice”) of individual veterinary clinical patient care were followed. The protocol of animal experimentation was reviewed and approved by the Ethics Review Board, in accordance with the standards recommended by the Guide for the Care and Use of Laboratory Animals and Directive 2010/63/EU for animal experiments. All animals were enrolled in the study after receiving the written consent of the owners, in compliance with the Italian Regulation D.L. 116/1992. 

### 2.2. Measurements

**Figure 1:**
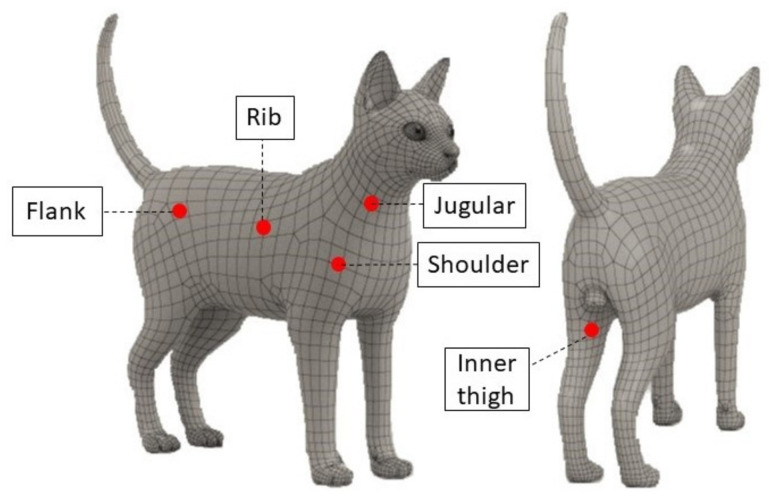
Graphical representation of measurement sites used for cutaneous temperature recording in cats. Jugular: the middle-third of the jugular vein; shoulder: the tip of the shoulder; rib: the middle-third of the chest, caudally to the heart area; flank: the dorsal portion of the flank, cranially to the iliac crest; inner thigh: caudally to the knee joint.

Cutaneous temperatures were recorded by means of three different non-contact infrared thermometers in five fully haired regions of interest: jugular, shoulder, rib, flank, and inner thigh (Figure 1). The measurement sites were standardized on the right side using anatomical landmarks as follows: jugular site at the middle-third of the jugular vein; shoulder site at the tip of the shoulder; rib site at the middle-third of the chest, caudally to the heart area; flank site at the dorsal portion of the flank, cranially to the iliac crest; and inner thigh site, caudally to the knee joint. 

The mentioned five regions of interest were chosen because animal handling is not required for reading the surface temperature in those areas [5].

The infrared thermometers used do not have a conversion factor incorporated, which converts a cutaneous reading into an estimated equivalent rectal reading.

The temperature and humidity conditions inside the experimental room were constantly monitored using a thermohygrometric probe. The microclimatic conditions were evaluated (mean ± standard deviation (SD)): the environment temperature was 20 ± 0.6 °C and the humidity was 47 ± 1.5%.

Prior to the use of infrared thermometers, the subjects were kept in the experimental room for approximately 15 min for acclimatization to the environmental conditions.

Three different infrared thermometers were used to assess the cutaneous temperature: IR1, the infrared thermometer Norditalia FI-150, with an accuracy of ±1.0 °C; IR2, the infrared thermometer THM010-VT001, Mediaid Inc., Cerritos, CA, USA, with an accuracy of ±0.3 °C; and IR3, the Fluke 62 mini-infrared thermometer, with an accuracy of ±1.5 °C. All the thermometers were placed at a distance of 5 cm from the measurement site. They were equipped with a tube placed above the sensor to reduce the sensor’s field of view (FOV) and to remove possible ambient IR sources. In total, 16 measurements were made for each cat. A single rectal temperature was measured using a digital thermometer (model HI92704, Hanna Instruments, Bedfordshire, UK), with a resolution of 0.1 ºC, that was inserted 3 cm into the rectum. 

The order of data recordings by means of the IR thermometers and the measurement sites were chosen randomly. The rectal temperature was evaluated after the cutaneous temperature to avoid false results because of temperature elevations due to activity and muscular exertion, resulting in a local temperature increase in the rectum [47].

All the cats enrolled in the study were accustomed to human presence and contact. During the experimental protocol, none of the cats showed behavioral disorders related to acute stress induced by cutaneous temperature measurement [32]. The same operator performed the temperature recordings.

To assess the accuracy of the digital and IR thermometers, we used them to evaluate the thermal recovery of an object preheated inside a thermostated oven, and then left it at room temperature for 15 min. A breast phantom, usually employed for quality controls of radiological equipment and made of material mimicking human tissue, was used.

All the temperature recordings were performed at the same time of the day (09:00) to avoid changes due to the circadian oscillation of the body temperature, as observed in diurnal animals [32]. Owned cats have been observed to show diurnal activity, with a higher amount of activity during the photophase than during the scotophase, as a consequence of the close relationship with humans, inducing an adaptation of their lifestyle to those of their owners [33].

### 2.3. Statistical Analysis

A Kruskal–Wallis test was applied to evaluate the consistency of the experimental data recorded during the evaluation of the preheated object for the 15 min of the thermal recovery.

The obtained data, expressed as mean ± SD, were normally distributed (Kolmogorov–Smirnov test, *p* > 0.05). Two-way analysis of variance (ANOVA) was applied to determine statistically significant effects of the thermometer and body region on the cutaneous temperature measured in cats. Tukey’s test was applied for post hoc comparison. For the comparison among the different IR devices and body temperature measurements, the percentage of paired readings, in which the rectal–body temperature differences were within the clinically accepted limit of ±0.5 °C, was determined. 

The correlation coefficient (r) between rectal temperature and the cutaneous temperature recorded with the means of the various used thermometers in each tested region was evaluated. Agreement between the body temperature measurements and the rectal temperature measurements, and among the different thermometers, were determined by the Bland–Altman method. Bias was defined as the mean difference between two methods, and the limits of agreement (LOA) were calculated as the bias ± 1.96 (SD), as previously reported [4,5,27]. A value of *p* <0.05 was considered statistically significant. The data were analyzed using the statistical software Prism v.5.00 (Graphpad Software Ltd., San Diego, CA, USA, 2003).

## 3. Results

The results of the measurements of an object preheated inside a thermostated oven and then left at room temperature for 15 min are presented in Table 1. The application of the Kruskal–Wallis test indicated no significant differences between the measurements obtained from the four different instruments (*p* = 0.98542, significance level *p* < 0.01). Having assessed the consistency of the probes, we proceeded with the experimental protocol on cats.

Table 2 reports the mean ± SD of the cutaneous temperature recorded in the five regions by means of the three different non-contact infrared thermometers and of rectal temperature recorded by a digital thermometer. The rectal temperature recorded in the 20 healthy cats was 38.59 ± 0.60 °C. The two-way ANOVA showed a significant effect of the different thermometers (*p* < 0.0001) and body regions (*p* < 0.0001) on the cutaneous temperatures in the cats. In particular, regarding the data recorded by the different thermometers, IR3 recorded a lower temperature than IR1 in all the body regions investigated (jugular *p* < 0.0001, shoulder *p* < 0.0001, rib *p* < 0.0001, flank *p* < 0.05, inner thigh *p* < 0.0001). IR3 recorded a lower temperature than IR2 in the jugular (*p* < 0.01) and shoulder (*p* < 0.01) regions. IR2 recorded a lower temperature than IR1 in the jugular (*p* < 0.0001), shoulder (*p* < 0.05), rib (*p* < 0.0001), and inner thigh (*p* < 0.05) regions. Regarding the various body regions investigated, IR1 gave a higher temperature in the inner thigh than in the jugular, rib, and flank (*p* < 0.01). IR3 recorded a lower value of the cutaneous temperature in the jugular region than in the shoulder (*p* < 0.01), rib (*p* < 0.01), flank (*p* < 0.0001), and inner thigh (*p* < 0.0001) regions; moreover, the temperature of the inner thigh region was higher than that in the shoulder region (*p* < 0.01). The use of IR2 did not show differences in the recording of the cutaneous temperature in the various regions studied.

The mean cutaneous temperature values from IR1, IR2, and IR3 tended to be lower than the rectal temperature by, respectively, 1.38 °C to 3.04 °C, 3.42 °C to 4.18 °C, and 4.51 °C to 7.08 °C. Out of 300 measurements, 3.43% of cutaneous temperature measurements by the three IR thermometers within ±0.5 °C of rectal temperature measurements was reported. 

The rectal temperature value recorded by means of digital thermometer was positively correlated with the cutaneous temperature recorded at the jugular (*p* < 0.05; r = 0.61), shoulder (*p* < 0.05; r = 0.66), and flank (*p* < 0.01; r = 0.75) regions with the IR1 thermometer.

**Figure 2:**
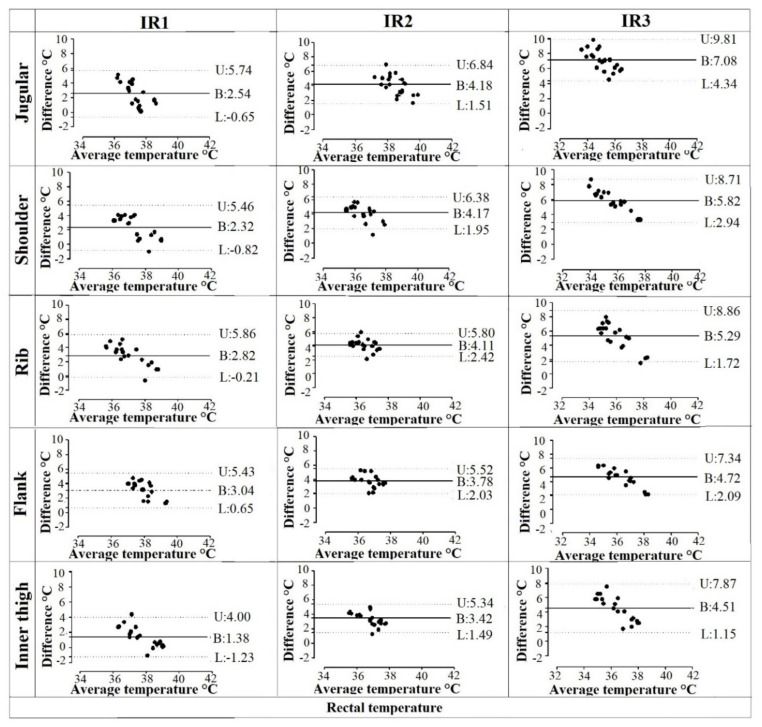
Bland–Altman comparisons of the rectal temperature values recorded with a digital thermometer, with the cutaneous temperature recorded using the three different non-contact infrared thermometers (IR1, IR2, and IR3) on the different cutaneous sites (jugular, shoulder, rib, flank, and inner thigh) in twenty healthy cats. The dotted lines represent the lower (L) and upper (U) limits of agreement (LOA); the solid line represents the mean difference (bias: B rectal temperature—cutaneous temperature) between the cutaneous and the rectal temperature measurements.

Agreement between the cutaneous and rectal temperature measurements was not satisfactory, as shown by the Bland–Altman plots (Figure 2), in which the mean temperature values were far from the zero-bias line, with a large SD of the bias and a large width of the limits of agreement. In cutaneous infrared measurements, the temperature was overestimated at lower values of the rectal temperature range, and underestimated at higher values.

Considering the comparison of the various IR thermometers used, with respect to the rectal one, the lowest bias was observed using the IR1 in all investigated regions; considering the various investigated body regions, the lowest bias was observed in the inner thigh (Table 3).

## 4. Discussion

In the current study, we compared three non-contact infrared thermometers with a digital rectal thermometer in the evaluation of body temperature in healthy cats by the investigation of five body regions. As expected, the comparison of the rectal and cutaneous temperatures showed a higher rectal temperature value than the cutaneous one. Although measurements of jugular, shoulder, and flank cutaneous temperature by only one IR thermometer correlated modestly with rectal temperatures, the agreement between the two methods was too poor to recommend the use of the tested infrared devices in clinical practice. 

The obtained results are in accordance with previous studies conducted with different strategies and infrared thermometers, and in different animal species [2,44].

Kunkle et al. [45] reported highly variable values using an infrared thermometer for the body temperature measurements (a mean difference of 0.07 °C and limits of agreement of 1.43 °C and −1.36 °C), which they considered unacceptable for clinical purposes in cats. 

According to the present results, it is not only the differences between the rectal and cutaneous temperature that make the use of infrared thermometers unacceptable for the monitoring of body temperature in cats, but also the fact that the infrared devices generated results that were not in good agreement among themselves. The obtained data were not reproducible and not constant. 

To date, despite the large existing literature on infrared methodology in veterinary medicine [2,3,4,5,6,7,8,16,18,19,22,23,24,28,29,30,31,32,33,34,35,36,37,38,39,40,41,42,43,44,45,46,47,48,49,50,51,52], no attention has been given to monitoring the performance of different infrared devices, comparing their surface temperature measurements in cats. In other species, the differences between rectal and cutaneous temperature have been established to be a conversion factor when the alternative method is used for the recording of body temperature instead of the gold standard of rectal temperature [29]. The infrared thermometers used do not have a conversion factor incorporated, which converts a cutaneous reading into an estimated equivalent rectal reading, strengthening the explanation that the detected differences among the various cutaneous sites and rectal temperature measurements were due to physiological temperature differences. 

The comparison of the cutaneous temperature measurements on the different body regions and by the three examined thermometers showed that the IR3 thermometer recorded lower mean cutaneous temperature values at the jugular region than at the shoulder, flank, rib, and inner thigh regions. 

The measurement values obtained with IR2 showed no statistically significant differences among them. IR1 recorded higher temperature values than all the cutaneous thermometers used in this study. The jugular, shoulder, and inner thigh showed different temperature values using all tools, whereas the rib and flank provided different values with IR3 as compared with IR1.

The differences observed among the recorded data may represent a physiological difference between body sites. The differences in the cutaneous temperature could be due to the relative power of the different body regions to emit heat by radiation (emissivity), linked to the length, color, and density of the coat, the thinner fur, and the muscle mass [36,37]. It is likely that the hair coat impeded infrared emission detection at some sites, which would explain why lower temperature readings were associated with a greater length of the coat [5,52]. The different arterial blood flows in each body portion represent another factor that can play a role in the variability of the data [5]. The same expert operator performed the temperature measurements, thus excluding the inter-rater variability. 

The assessed regions showed different cutaneous temperature values, also regarding the infrared methodology used. The three IR methodologies showed low repeatability of data recordings in vivo, in contrast to the high repeatability observed during the test performed on the heated object before the start of the study. This finding is in agreement with previously reported findings in feline species regarding the use of tympanic infrared thermometers [7]. However, the reason for this difference is not clear and needs further investigation.

In an ideal instrument, the difference between infrared and rectal thermometry would be 0 °C at any temperature measured [27]. Statistical analysis showed poor agreement among the cutaneous thermometry data with wide limits of agreement and large bias, confirming that cutaneous temperature measurement in the studied body regions cannot be used as a reliable substitute for rectal temperature measurement in clinical settings. Moreover, the fact that less than 4% of cutaneous temperature measurements in different body sites and with different devices were not within ±0.5 °C of rectal temperatures suggests that these methods should not be used interchangeably in cats. The poor interchangeability is also due to the fact that the infrared devices generated results not in good agreement with them, despite their similar technical characteristics.

In addition to the only moderate correlation between methods, all of the cutaneous-rectal temperature Bland–Altman plots showed variable bias, with generally positive temperature differences at low average body temperatures, and generally negative temperature differences at high average body temperatures, suggesting perhaps that surface temperature remains relatively constant despite changes in rectal temperature. This finding further underlines how the cutaneous temperature differs from the rectal one. 

Our results are striking in that we observed this low concordance between rectal and infrared temperatures in healthy cats maintained in a zone of thermal neutrality at standardized external conditions to reduce the effects of external variables. Infrared temperature can be expected to state more than 1° C (until about 7 °C) below a rectal temperature, and this discrepancy precludes the use of infrared thermometry in feline practice. 

As reported in hypo- and hyperthermic cats [5] and in young febrile children [26], this study showed a significant device overestimation of infrared temperature at low rectal temperatures and an underestimation at elevated rectal temperatures also in normothermic cats.

The present study has some limitations, such as the small sample size and the lack of hypothermic and hyperthermic measurements to permit sensitivity/specificity testing to detect abnormal body temperatures. Although the small sample size may be warranted by the narrow inclusion criteria necessary to obtain a homogeneous sample of healthy cats, further studies are needed on a larger number of cats to confirm our results in different environmental conditions and in hypo/hyperthermic subjects. 

## 5. Conclusions

This study demonstrated that infrared temperature measurement devices generated results that were not in good agreement among themselves, providing a novel result of clinical importance. In the present study, infrared thermometry used in the region of interest of the jugular, shoulder, rib, flank, and inner thigh does not correlate to rectal temperature. So, the cutaneous temperature does not seem to be a reliable alternative to the rectal temperature for measuring the body temperature in cats. In cats, the use of non-contact infrared thermometers gave no reproducible or constant data to justify their application for the recording of body temperature instead of rectal temperature recording. In addition, the absence of reproducible data prevented the establishment of the normal cutaneous temperature range in the various body regions investigated.

## Figures and Tables

**Table 1:** Temperature (°C) recorded for the accuracy assessment of the different thermometers used during the experimental protocol. The temperature of a preheated object was recorded for 15 min during its thermal recovery every 1 min by means of digital and infrared (IR) thermometers.

Minutes	Digital	IR1	IR2	IR3
0	42	42	42	41.8
1	40.8	40.7	40.6	40.7
2	40.1	40	40	39.8
3	39.2	39.1	39.2	38.8
4	38.2	38.4	38.4	38
5	37.5	37.2	37.3	37.1
6	36.4	36.1	36	35.9
7	35.6	35.2	35.3	35
8	34.8	34.5	34.6	34.5
9	34	33.8	33.7	33.6
10	32.9	33	32.8	32.8
11	32	31.6	31.7	31.8
12	29.8	29.7	29.8	29.8
13	29.3	28.9	29	29.1
14	28.5	28.2	28.4	28.3
15	27.3	27.2	27.3	27.2

**Table 2:** Means ± SD of cutaneous temperature recorded in the five body regions (jugular, shoulder, rib, flank, inner thigh) obtained with the three different thermometers, and rectal temperature recorded by a digital thermometer in twenty healthy cats, expressed in °C. Statistical significance is also reported. Symbols (^#^ = vs. jugular region, * = vs. shoulder region, ° = vs. rib and flank) indicate statistically differences of cutaneous temperature between the five different body regions (jugular, shoulder, rib, flank, inner thigh) using the same thermometer. Lower letters (^a^ = vs. IR1 and IR2, ^b^ = vs. IR1) indicate statistically different cutaneous temperature values recorded in the same cutaneous region using different thermometers (IR1, IR2, IR3).

Thermometers	Jugular	Shoulder	Rib	Flank	Inner thigh	Rectal
IR1	36.05 ± 1.39	36.27 ± 1.64	35.77 ± 1.73	35.55 ± 1.46	37.21 ± 1.54 ^#^°	
IR2	34.41 ± 1.25 ^b^	34.42 ± 1.70 ^b^	34.48 ± 0.87 ^b^	34.81 ± 0.89	35.17 ± 1.06 ^b^	
IR3	31.51 ± 1.42 ^a^	32.76 ± 1.93 ^a#^	33.30 ± 1.948 ^b#^	33.87 ± 1.80 ^b#^	34.08 ± 1.93 ^a#^*	
Digital						38.59 ± 0.60

**Table 3:** Bland–Altman comparisons of the cutaneous temperature recorded (the jugular, shoulder, rib, flank, and inner thigh) with three different non-contact infrared thermometers (IR1, IR2, and IR3) in twenty healthy cats.

Cutaneous Regions		Infrared Thermometers	IR2	IR3		IR3
Jugular	Upper Limit	IR1	5.66	8.40	IR2	6.96
	Bias		1.63	4.53		2.89
	Lower Limit		−2.38	0.66		−1.17
Shoulder	Upper Limit	IR1	3.94	7.29	IR2	5.10
	Bias		1.85	3.50		1.65
	Lower Limit		−0.24	−0.27		−1.78
Rib	Upper Limit	IR1	3.89	7.34	IR2	4.84
	Bias		1.29	2.47		1.18
	Lower Limit		−1.31	−2.40		−2.48
Flank	Upper Limit	IR1	2.94	4.38	IR2	3.57
	Bias		0.73	1.67		0.94
	Lower Limit		−1.47	−1.03		−1.69
Inner Thigh	Upper Limit	IR1	4.54	5.96	IR2	3.83
	Bias		2.03	3.13		1.09
	Lower Limit		0.46	0.29		−1.64

## Data Availability

The data that support the findings of this study are available on request from the corresponding author.
